# The positive correlation between physical exercise and the fertility intention of women of childbearing age: an empirical analysis based on the Chinese General Social Survey

**DOI:** 10.3389/fpubh.2026.1927726

**Published:** 2026-10-27

**Authors:** Mengmeng Man, Kehan Zhang, Chenlei Zhao, Yuchi Chu, Junhua Zhou

**Affiliations:** 1 School of Physical Education, Ludong University, Yantai, China; 2 School of Sports Economics and Management, Tianjin University of Sport, Tianjin, China; 3 Shandong Urban Service Vocational College, Yantai, China

**Keywords:** fertility intention, health status, mediating effect, physical exercise, subjective well-being

## Abstract

In the context of declining fertility rates and an aging population, enhancing the willingness to conceive among women of childbearing age is crucial for alleviating demographic challenges. This study analyzes data from the 2021 Chinese General Social Survey (CGSS) Using benchmark regression, propensity score matching, mediation analysis, and heterogeneity testing, this study examines the positive association between physical exercise and fertility intention. The findings indicate a statistically significant positive correlation. First, physical exercise is positively associated with ideal fertility among women of childbearing age. The Ordinary Least Squares (OLS) benchmark regression yielded a coefficient of 0.046 (*p* < 0.01) without controls and 0.087 (*p* < 0.01) with full control—results that were corroborated by multiple robustness checks. Second, mediation analysis indicates that health status and subjective well-being partially mediate this relationship: physical exercise is positively associated with ideal fertility by improving both health status and subjective well-being. Third, heterogeneity analysis shows that the coefficient estimates are descriptively larger for younger women (≤30) and urban residents, but formal interaction tests indicate that these differences are not statistically significant at conventional levels (*p* > 0.05). These findings suggest that population policies might consider physical exercise as a component, foster a fertility-friendly environment, and promote the reciprocal benefits of physical exercise and personal well-being, provided that future causal evidence supports the observed associations.

## 1 Introduction

Since the mid-20th century, global fertility rates have experienced a sustained decline following a brief postwar peak. The Organization for Economic Co-operation and Development (OECD) data indicate that the total fertility rate among its 38 member countries dropped from 3.3 in 1960 to 1.5 in 2022, with some nations now confronting ultra-low fertility. Vollset et al. (1) project that by 2100, 183 countries will have fertility rates below replacement level, suggesting that low fertility may become a global norm. According to China’s Seventh National Census, the total fertility rate has fallen to 1.3, reflecting a pronounced low-fertility trajectory. The National Bureau of Statistics reported only 9.02 million births in 2023, with a natural population growth rate of −1.48 and a total fertility rate hovering around 1.0—marking the second consecutive year of negative growth and placing China second-lowest among major global economies in this regard (2). Despite the implementation of the “three-child policy” and accompanying supportive measures in 2021, China continues to face persistent fertility decline and limited effectiveness of existing pro-natalist policies, underscoring the urgent need for innovative solutions.

Understanding the fertility intentions of women of childbearing age is essential for addressing the low-fertility challenge. These intentions are increasingly heterogeneous and serve as a critical predictor of actual fertility behavior. Rising educational attainment and labor force participation have shortened women’s effective reproductive windows while delaying average childbearing age. Fertility decision-making has shifted from a focus on the “quantity of children” toward more fundamental considerations of “whether to have children at all.” In this context, sole reliance on economic incentives or policy relaxation appears insufficient, highlighting the need to examine non-economic factors that may bolster fertility intentions.

Physical exercise, as an integral component of an active lifestyle, has demonstrated social value beyond its established health benefits. Regular exercise allows individuals to experience greater excitement and pleasure, alleviate negative emotions, and enhance physical fitness (3). Activities such as hiking, yoga, and jogging have become essential means for modern women to relieve stress and improve quality of life. Physical exercise not only contributes to women’s agency in health management but also strengthens self-identity, self-awareness, and the spiritual and cultural valuation of the body (4). This raises a core research question: Can physical exercise positively affect women’s fertility intentions by enhancing their physical and mental health? Theoretically, physical exercise has been shown to significantly improve individual health and subjective well-being, both of which are psychological resources influencing fertility decisions. However, few existing studies integrate physical exercise, health status, subjective well-being, and fertility intention within a unified analytical framework, and even fewer examine how this mechanism varies across demographic subgroups.

Women are central to social development, and their fertility status exerts a decisive influence on overall fertility rates. This study draws on the second demographic transition theory and data from the 2021 Chinese General Social Survey (CGSS2021) to empirically investigate how physical exercise affects the fertility intentions of women of childbearing age. Using correlation analysis, benchmark regression models, and mediation analysis, we address the following questions:

1. Is physical exercise associated with higher fertility intention among women of childbearing age?
2. Through what mechanisms does physical exercise exert its influence?
3. How do these effects vary across age groups and urban–rural residence?

The findings aim to provide scientific evidence for designing effective fertility support policies that enhance fertility intentions and contribute to a balanced demographic trajectory.

### 1.1 Literature review and hypothesis development

Fertility intentions among women of childbearing age are shaped by a combination of economic and non-economic factors. Economic determinants include population mobility, socioeconomic status, and access to social security (5), while non-economic factors encompass individual-level dimensions such as health, mental well-being, and subjective happiness—all of which can significantly enhance fertility intention. Demographic research has often overlooked the specific role of physical exercise in shaping fertility intention, instead attributing it to broader socioeconomic and psychological conditions. Despite the time and financial costs that may compete with parenting responsibilities (6), physical exercise serves as an important non-economic factor that alleviates stress, improves physical health, and is positively associated with quality of life. These benefits can positively influence psychological well-being and family relationships, thereby directly or indirectly strengthening fertility intention.

In 1986, Lesthaeghe and Van de Kaa proposed the second demographic transition theory to explain declining fertility and shifting family norms in Europe (7, 8). This theory links fertility decline to changes in personal consciousness, including the rise of individualism and feminism, the strengthening of women’s self-pursuit, evolving gender roles, and the pursuit of personal value and self-actualization. In China, changing social dynamics—smaller families, increased mobility, and reduced traditional family hierarchies and parent–child concepts—are accelerating this transition. These changes have led women of childbearing age to place greater emphasis on individual needs and experiences, including the importance of physical exercise. Child needs theory suggests that parenting fulfills psychological and emotional needs, benefiting both individuals and families (9). Thus, physical exercise, as an individual need that does not necessarily compete with traditional family relationships (10), can meet the physical and mental development needs of women, enhance physical health, and increase subjective well-being, thereby strengthening fertility intention. Luppi and Mencarini reported that lack of enjoyment in physical exercise correlates with lower fertility intentions (11).

Women’s engagement in physical exercise is closely linked to human development. Based on human development theory, Coall et al. (12) argue that individuals increasingly prioritize their own needs over time and seek to balance reproduction and psychological well-being. Without guaranteed access to recreational activities—including physical exercise that supports reproductive health—women’s willingness to have children may diminish. Iwasaki (13) found that physical exercise is a crucial means for women of childbearing age to manage fatigue and stress, offering protective benefits in high-stress situations. Jarosz et al. (14) reported that physical exercise alleviates work-related fatigue, improves overall health, and provides physical and psychological rewards while meeting personal goals or lifestyle needs. Shahid and Noor (15) demonstrated that testosterone levels rise immediately after exercise but do not remain elevated for extended periods, often exhibiting a rebound effect; exercise volume significantly affects post-exercise testosterone, which in turn regulates muscle strength and bone mineral density in women, thereby promoting strength, cognition, and sexual function and increasing fertility intention. Lin et al. analyzing data from the 2022 China Family Panel Studies (CFPS), found that for each additional unit of physical engagement, the probability of fertility among married young adults increases by 0.048—an effect achieved through optimization of family system functioning. Physical exercise satisfies three basic psychological needs: autonomy, competence, and belonging, thereby enhancing confidence and readiness for the reproductive role (16). Similarly, an empirical study by Bu et al. (17) using 2023 CGSS data confirmed that physical exercise significantly and positively affects young people’s fertility attitudes through two pathways: improving physical and mental health (health effect) and enhancing family cohesion (harmony effect), with the health effect playing a particularly prominent mediating role. Kandel et al. (18) mixed-method study revealed multiple motivations for preconception physical exercise, including health pursuit, weight management, social support, and personal goal setting, which collectively create the psychosocial foundation for exercise to promote reproductive health.

While recent scholarship has investigated the link between physical exercise and fertility-related outcomes, this study delivers several distinct contributions relative to closely related work by Bu et al. (17), Lin et al. (16), and Deng et al. (19). Bu et al. (17) examined fertility attitudes among mixed-gender young adults with health and family harmony as mediators; in contrast, our analysis focuses specifically on fertility intention among women of reproductive age. Lin et al. (16) and Deng et al. (19) employed sequential mediation centered on family functioning and marital satisfaction, whereas we adopt a parallel mediation framework for health status and subjective well-being. This setup allows us to disentangle their independent indirect effects and assess whether the two pathways are complementary or redundant, a question unaddressed by sequential mediation models. Additionally, we apply multiple propensity score matching estimators alongside Rosenbaum bound sensitivity analysis, providing a more robust assessment of selection bias than prior single-model designs. We note these are incremental rather than revolutionary contributions. We do not introduce a new identification strategy or theoretical framework, but offer a methodologically transparent, female-specific estimate of the exercise–fertility intention association to complement earlier evidence from broader samples and alternative mediation specifications.

Thus, we propose the first theoretical hypothesis:

*H1*: Physical exercise is positively associated with ideal fertility among women of reproductive age.

Physical exercise affects the physical condition of women during the reproductive process. Regular exercise is positively associated with physiological functions and improves overall health (20), which supports reproductive capacity. From a medical perspective, exercise contributes to improved physical well-being. Suntornsaratoon et al. reported that low calcium intake combined with physical exercise correlates with trabecular and cortical thickness, improves skeletal muscle function, and helps prevent bone deficiencies—thereby improving health levels (21). Although women select different types of physical exercise according to personal values, any form of exercise can positively impact health. Ye et al. (22) using data from the National Health and Nutrition Examination Survey (NHANES) for 2013–2018, found that women following a healthy lifestyle—including engaging in at least 150 min of moderate-to-vigorous physical exercise per week—had a significantly reduced risk of infertility. Specifically, women adhering to 4–5 healthy lifestyle factors (healthy diet, moderate alcohol consumption, normal waist circumference, adequate physical exercise, and non-smoking) experienced a 59% lower risk of infertility compared to those with only 0–1 healthy lifestyle factors (OR = 0.41, 95% CI: 0.23–0.76). Furthermore, for every one-point increase in the healthy lifestyle score, the risk of infertility decreased by 21% (OR = 0.79, 95% CI: 0.68–0.92). Fertile women exhibited significantly higher levels of physical exercise than infertile women, and women who sat for more than 300 min per day had a 2.07 times higher risk of fertility problems than moderately active women (22). Zborowska et al. (23) found that postpartum women using EMS technology for functional training experienced significant improvements in pelvic floor muscle function, sexual quality and satisfaction, and overall health and body self-evaluation—all essential components of reproductive health. A prospective cohort study (SF/Activity) at Aarhus University is further exploring causal effects of pre-pregnancy and early-pregnancy exercise on fertility, spontaneous abortion, and pregnancy outcomes, aiming to provide higher-level evidence on the dose–response relationship between physical exercise and female reproductive health (24, 25).

Drawing on Grossman’s health capital theory (26) and integrating findings from modern epidemiological and physiological mechanism studies, there is multi-source evidence that physical exercise correlates with the health status of women of childbearing age. It improves their reproductive physiological foundation while boosting confidence and readiness for reproductive roles, thereby increasing willingness to have children.

Therefore, we propose a second theoretical hypothesis:

*H2*: Physical exercise is associated with better health, which in turn is associated with higher ideal fertility.

Physical exercise can also enhance subjective well-being, which subsequently influences fertility desires. Regular exercise improves not only physical health but also psychological well-being and overall life satisfaction (25). Chen et al. (27) demonstrated that physical exercise has a strong antidepressant effect and can positively impact mental health by modulating amygdala responses to stress, validating its clinical use as anti-anxiety therapy. As a key contributor to psychological capital, physical exercise significantly boosts mental health and is related to subjective well-being. Ruseski et al. (28) reported that regular exercise correlates with social capital, further improving well-being. Several scholars have explored the link between subjective well-being and fertility intention. Leibenstein reported that the benefits of having children are primarily manifested as a profound sense of fulfillment, encouraging childbearing when anticipated well-being outweighs economic costs (10). Ajzen et al. (29) also showed that individuals with positive attitudes toward fertility and expectations of happiness through childbearing generally exhibit stronger willingness to have children, ultimately leading to higher fertility rates.

Recent empirical studies robustly support the pathway through which physical exercise augments fertility by enhancing subjective well-being. An integrated analysis by Deng et al. based on CGSS data for 2017, 2018, and 2021, revealed that physical exercise improves fertility by raising marital satisfaction and subjective well-being among young married individuals—an effect persisting after controlling for endogeneity (19). Furthermore, Bu et al. (17) found that physical exercise influences fertility attitudes among youth via the “harmony effect,” underscoring the crucial role of subjective well-being in linking exercise with fertility intentions. Mazloomy et al. (30) using path analysis grounded in self-determination theory with 412 women of childbearing age, demonstrated that autonomous perception, enjoyment, health motivation, and social motivation indirectly affect physical exercise behavior through the relative autonomy index. When exercise satisfies an individual’s needs for autonomy, competence, and belonging, it provokes a more enduring sensation of happiness, fostering positive expectations toward the reproductive role. Fallahi et al. (31) cross-sectional study of 483 infertile women found that both social support and self-regulation significantly influenced changes in exercise behavior (OR = 1.05, 95% CI: 1.02–1.09; OR = 1.06, 95% CI: 1.02–1.11), indicating that exercise correlates with an individual’s sense of belonging and emotional connection through social support networks, thereby increasing subjective well-being—a cumulative social psychological resource that can boost confidence and willingness to have children.

In conclusion, physical exercise can effectively enhance fertility intentions among women of childbearing age, whether through direct positive emotional experiences or via indirect pathways such as family harmony and social support networks.

Hence, we propose a third research hypothesis:

*H3*: The association between physical exercise and ideal fertility is statistically accounted for in part by subjective well-being.

## 2 Materials and methods

### 2.1 Data sources

The data for this study are drawn from the CGSS2021, conducted by Renmin University of China and publicly released on March 31, 2023. The 14th annual Chinese General Social Survey was conducted in collaboration with a survey alliance of cooperating universities across all Chinese provinces and municipalities directly under the central government, with the fieldwork completed over approximately 5 months. The survey covered 21 provinces, autonomous regions, and municipalities directly under the central government. The CGSS employs a stratified four-stage probability sampling design covering societal, community, household, and individual levels. The questionnaire structure is well-constructed, and the sampling validity and reliability have been verified by the China Survey and Data Center. The final analytical sample consisted of 1,230 participants. This study targeted women within the typical reproductive-age range of 20–50 years. Starting from an initial dataset of 8,148 respondents, we sequentially excluded male participants (remaining = 4,469), individuals with divorced or non-standard marital status (remaining = 3,811), and subjects outside the 20-50-year age bracket (remaining = 1,586). Observations with ambiguous responses on fertility intention and physical-exercise frequency were further dropped, yielding the final sample of 1,230 individuals. All statistical analyses were performed using Stata (version 17.0).

### 2.2 Variable selection

#### 2.2.1 Dependent variable: fertility intention

The dependent variable was measured using the CGSS2021 questionnaire item A37-1: “In the absence of policy restrictions, how many children would you like to have?” Responses included desired number of sons and daughters, which were summed to yield a total fertility intention score ranging from 0 to 10. For data consistency, responses greater than 6 were recoded to 6, given the rarity of such cases. We acknowledge that this measure captures “ideal” rather than “planned” fertility, and we address this limitation in the Discussion.

#### 2.2.2 Independent variable: physical exercise

Physical exercise was measured using question A30-9: “How often did you engage in physical exercise in your spare time over the past year?” Response options were: “Never” (coded 0), “Several times a year or less” (coded 1), “Several times a month” (coded 2), “Several times a week” (coded 3), and “Daily” (coded 4). This ordinal measure was used in the main analyses; for the PSM robustness check, it was dichotomized into exercise (≥1 time) versus no exercise (0), as detailed below.

#### 2.2.3 Mediating variables: health status and subjective well-being

Health status was assessed using self-rated health (item A15) on a 5-point scale from “Very unhealthy” (1) to “Very healthy” (5). Subjective well-being was measured using item A36 on a 5-point scale from “Very unhappy” (1) to “Very happy” (5).

#### 2.2.4 Control variables

Based on Wesolowski (32), we selected control variables at the individual, family, and social levels. Individual-level factors included age, household registration (urban vs. rural), and educational attainment; family-level factors included marital status; social-level factors included social frequency, social interaction, social class, and socioeconomic status. Table 1 presents descriptive statistics for all variables.

**Table 1:** Variable names, definitions, and descriptive statistics.

Variable names	Source item	Variable definitions	Mean	SD
Dependent variable	Fertility intention A37–1	Propensity to have children 0–6	1.751	0.745
Independent variables	Physical exercise A30–9	Never = 0, several times a year or less = 1, several times a month = 2, several times a week = 3, daily = 4	1.840	1.423
Mediating variable	Health status A15	Very unhealthy = 1, relatively unhealthy = 2, average = 3, relatively healthy = 4, very healthy = 5	3.905	0.891
	Subjective well-being A36	Very unhappy = 1, relatively unhappy = 2, neither happy nor unhappy = 3, relatively happy = 4, very happy = 5	4.015	0.693
Control variables	Age A3–1	Age	36.115	8.923
	Household registration A18	Urban residents = 1, rural residents = 0	0.332	0.471
	Education level A7a	Primary school and below = 1, junior high school = 2, senior high school (technical secondary school) = 3, junior college = 4, bachelor’s degree and above = 5	2.996	1.434
	Marital status A69	Married = 1, not married = 0	0.735	0.442
	Social frequency A31-1	Never = 1, rarely = 2, sometimes = 3, often = 4, frequently = 5	2.734	0.988
	Social interaction A31b	Never = 1, once a year or less = 2, several times a year = 3, about once a month = 4, several times a month = 5, once to twice a week = 6, every day = 7	4.110	1.714
	Social class A43-a	1–2 points = lower (1), 3–4 points = middle lower (2), 5–6 points = middle (3), 7–8 points = middle upper (4), 9–10 points = upper (5)	2.545	0.832
	Socioeconomic status A43e	Lower class = 1, lower middle class = 2, middle class = 3, upper middle class = 4, upper class = 5	2.354	0.812

### 2.3 Evaluation of the rationality of sample size

Prospective sample size calculations for OLS regression models were performed using GPower 3.1 software. Based on existing empirical studies examining the relationship between physical exercise and fertility intention (19), the effect size, Cohen’s *f*^2^, was determined to be 0.03 (a small-to-medium effect, consistent with non-economic factors on fertility intention in social sciences). With significance level *α* = 0.05 and the statistical power 1 − β = 0.80, and including eight control variables, the minimum required sample size was 680. Our final sample of 1,230 valid observations substantially exceeds this threshold, ensuring adequate statistical power to detect the hypothesized effects. Moreover, the CGSS2021 employs a nationally representative sample, and the selected 1,230 women of childbearing age are broadly consistent with national population distribution in terms of age, household registration, and educational level, supporting the external validity of our findings. Compared with the CGSS2021 population distribution, our analytic sample has similar age composition (mean 36.1 vs. 36.0 in full sample) and urban–rural ratio (33.2% urban vs. 33.0%), supporting generalizability.

### 2.4 Data analysis strategies

This study employed quantitative analysis methods with the following steps: First, descriptive statistics were computed to characterize the sample distribution. Second, a benchmark regression model tested the total effect of physical exercise on fertility intention, and variance inflation factor (VIF) diagnostics were used to examine multicollinearity among explanatory variables. Third, a mediation model was constructed following the stepwise approach of Baron and Kenny (33), with indirect effect significance evaluated using the Bootstrap method with 1,000 resamplings (33). Fourth, robustness tests were conducted through propensity score matching (PSM) and alternative model specifications (Ordered Probit). Fifth, heterogeneity analyses by age group and household registration were performed using interaction-term specifications rather than separate subsample comparisons to formally test coefficient differences. These steps comprehensively reveal the effects, mechanisms, and group differences in the relationship between physical exercise and fertility intentions.

### 2.5 Benchmark regression model

Our dependent variable, fertility intention, ranges from 0 to 6 and is derived by summing desired sons and daughters. While this is technically a count variable, we primarily employed OLS regression for three reasons: (1) the variable spans 7 ordered levels with a distribution approximating continuous data; (2) OLS coefficients directly quantify the absolute increase in fertility intention per unit increase in physical exercise, facilitating substantive interpretation; and (3) OLS maintains consistency across main and robustness analyses. To address potential heteroscedasticity, all OLS models were estimated with heteroscedasticity-robust standard errors. We also report Ordered Probit results as a robustness check, which confirm the main findings.

The benchmark model is specified in Equation 1.


$$\mathrm{FI}=\gamma_{1}\mathrm{PE}+\gamma_{2}\text{control}+\alpha_{0}+\varepsilon_{\mathrm{it}}$$ (1)

where FI represents fertility intention, PE represents physical exercise, Control represents the vector of control variables, γ is the coefficient of interest, α₀ is the intercept, and ε is the random error term.

### 2.6 Mediating effect model

To clarify the micro-mechanisms through which physical exercise affects fertility intention, we constructed a stepwise mediation model with health status and subjective well-being as mediators, as shown in Equations 2–4:


$$\mathrm{FI}=\gamma_{1}\mathrm{PE}+\gamma_{2}\text{control}+\alpha_{0}+\varepsilon_{\mathrm{it}}$$ (2)

$$M=\gamma_{1}\mathrm{PE}+\gamma_{2}\text{control}+\alpha_{0}+\varepsilon_{\mathrm{it}}$$ (3)

$$\mathrm{FI}=\gamma_{1}\mathrm{PE}+\gamma_{2}M+\gamma_{3}\text{control}+\alpha_{0}+\varepsilon_{\mathrm{it}}$$ (4)

where *M* is the mediating variable health status and subjective well-being, to investigate whether health status and subjective well-being have a mediating effect on the impact of physical exercise on the fertility intention of women of childbearing age, the first step was to examine the impact of physical exercise on the fertility intention of women of childbearing age, and the second step was to investigate the impact of physical exercise on health status and subjective well-being. The third step was to investigate the impact of physical exercise on health and subjective well-being on fertility intentions.

## 3 Results

### 3.1 Correlation analysis

Table 2 presents Pearson correlation coefficients. The independent variable, physical exercise, is positively and significantly correlated with fertility intention (*r* = 0.088, *p* < 0.05). All correlation coefficients are below 0.7, indicating no serious multicollinearity concerns. However, bivariate correlations do not account for confounding variables; thus, multivariate regression analyses are necessary for robust inference.

**Table 2:** Correlation analysis table.

Variables	Fertility intentions	Physical exercise	Age	Marital status	Education level	Social frequency	Social class
Fertility intentions	1						
Physical exercise	0.088***	1					
Age	0.286***	−0.0300	1				
Marital status	0.260***	0.090***	0.525***	1			
Education level	0.263***	0.300***	0.495***	0.446***	1		
Social frequency	−0.00800	0.092***	0.118***	0.072 * *	0.112***	1	
Social class	−0.00400	0.138***	0.074***	−0.0300	0.198***	0.094***	1
Social interaction	0.106***	0.196***	0.239***	0.231***	0.267***	0.393***	0.151***
Socioeconomic status	0.00200	0.089***	−0.0200	0.0140	0.123***	0.077***	0.537***
Household registration	0.152***	0.202***	0.063 * *	0.156***	0.416***	0.0180	0.147***

***p < 0.01, **p < 0.05, *p < 0.1.

### 3.2 Multicollinearity test

As shown in Table 3, VIF values for all explanatory variables range from 1.16 to 1.90 (mean VIF = 1.43), well below the conventional threshold of 5, indicating no serious multicollinearity. Tolerance values (1/VIF) are all close to 1, confirming that the estimation assumptions of the benchmark regression model are satisfied.

**Table 3:** Multicollinearity test (VIF).

Variable	VIF	Tolerance (1/VIF)
Physical exercise	1.16	0.865
Age	1.66	0.602
Marital status	1.50	0.667
Education level	1.90	0.528
Social frequency	1.19	0.840
Social class	1.46	0.686
Social interaction	1.32	0.758
Socioeconomic status	1.42	0.706
Household registration	1.27	0.787
Mean	1.43	—

VIF>10 indicates severe multicollinearity; VIF>5 indicates strong multicollinearity.

### 3.3 Benchmark regression analysis

We constructed two OLS regression models to examine the impact of physical exercise on fertility intentions. Model 1 excluded control variables to estimate the unadjusted association, while Model 2 incorporated the full set of individual-, family-, and social-level controls to isolate the net effect of physical exercise. Table 4 presents the results.

**Table 4:** Benchmark regression results.

Variables	OLS (1)	OLS (2)
	Fertility intentions	Fertility intention
Physical exercise	0.046***	0.087***
	(3.079)	(5.849)
Age		0.013***
		(4.441)
Marital status		0.180***
		(3.284)
Education level		−0.077***
		(−4.076)
Social frequency		0.026
		(1.201)
Social class		0.029
		(1.002)
Social interaction		−0.019
		(−1.457)
Socioeconomic status		0.004
		(0.129)
Household registration		−0.154***
		(−3.263)
Constant	1.667***	1.210***
	(48.163)	(7.889)
Observations	1,230	1,230
*R*-squared	0.008	0.144
r2_a	0.00685	0.137
*F*	9.480	22.77

*t*-statistics in parentheses; robust standard errors; ***p < 0.01, **p < 0.05, *p < 0.1.

Model 1 (without controls) shows a positive and significant effect of physical exercise on fertility intention (coefficient = 0.046, *t* = 3.079, *p* < 0.01), providing preliminary support for Hypothesis H1. Model 2 (with full controls) yields a substantially larger coefficient (0.087, *t* = 5.849, *p* < 0.01), nearly double that of Model 1. Given the low VIFs documented in Section 3.2 (all <1.90, mean = 1.43), this amplification is unlikely to be an artifact of multicollinearity or predictor redundancy. Instead, we interpret this increase as a model specification gain attributable to the inclusion of suppression-like confounders. Specifically, education level and household registration exhibit positive bivariate correlations with both physical exercise and fertility intention, yet their conditional net effects in Model 2 turn significantly negative (−0.077 and −0.154, respectively) once age and marital status are held constant. This pattern suggests that these variables functioned as masking factors in the bivariate specification: their positive simple correlations with fertility intention were positively confounded by their association with younger, unmarried subsamples, inadvertently obscuring the net association of physical exercise. After accounting for these conditional effects, the intrinsic positive association between exercise and fertility intention emerges more fully. Importantly, because the VIF diagnostics rule out multicollinearity (Section 3.2), this coefficient expansion can be plausibly attributed to the correction of omitted-variable bias rather than statistical overlap among predictors.

Among the control variables, age (0.013, *p* < 0.01), marital status (0.180, *p* < 0.01), and household registration (−0.154, *p* < 0.01) show significant associations in expected directions, while education level exerts a significant negative effect (−0.077, *p* < 0.01), consistent with the well-documented education-fertility trade-off. Social-level variables (social frequency, social class, social interaction, and socioeconomic status) do not reach statistical significance (*p* > 0.1). Their VIFs, all below 1.46, suggest that these null findings are not attributable to collinearity with other covariates; rather, their influence on fertility intention may be more distal, operating indirectly through individual-level psychological mediators not captured in the current parsimonious model.

Model fit improves substantially with the inclusion of controls, with *R*^2^ increasing from 0.8 to 14.4% (adjusted *R*^2^ from 0.69 to 13.7%), and the *F*-statistic rising from 9.48 to 22.77, confirming that the full set of covariates jointly contributes significantly to explaining variation in fertility intention. Importantly, the coefficient for physical exercise remains highly significant after controls are added, and combined with the VIF evidence ruling out confounding distortions (Section 3.2), this reinforces our confidence that the positive association is robust and not an artifact of omitted variable bias. These results provide strong support for Hypothesis H1.

### 3.4 Robustness test

To address concerns about sample selection bias and model specification, we conducted two sets of robustness checks: propensity score matching (PSM) (34) to mitigate observable selection bias and Ordered Probit estimation to rule out functional form misspecification. Although our benchmark regression included a comprehensive set of control variables, the possibility remains that women who choose to exercise differ systematically from those who do not on observable characteristics that also affect fertility intentions. To reduce this selection bias, we employed 1:1 nearest-neighbor matching with a caliper of 0.05, using all baseline control variables as matching covariates. Physical exercise was redefined as a binary treatment variable for this analysis: women who exercised at least once were assigned to the treatment group (coded 1), while those who never exercised formed the control group (coded 0).

We first performed 1:1 nearest-neighbor matching with a caliper of 0.05. As shown in Table 5, the matching successfully eliminated significant covariate imbalances; standardized mean differences were reduced to below 10%, and t-tests confirmed no significant differences between groups (*p* > 0.05 for all matched variables). Regression on this matched sample (*N* = 429) yielded a positive coefficient of 0.069 (*t* = 2.961, *p* < 0.01), confirming the benchmark finding. However, this specification discards many untreated observations to achieve tight pairwise balance, which may reduce estimation efficiency.

**Table 5:** Results of the balance test.

Variables	Before and after matching	Mean values of the experimental group	Control group mean	Standardized deviation	*T* value	*p*-value
Age	U	35.565	37.681	−24.000	−3.670	0.000
	M	35.126	35.224	−1.100	−0.220	0.828
Marital status	U	0.701	0.831	−31.100	−4.570	0.000
	M	0.689	0.706	−4.000	−0.740	0.457
Education level	U	3.326	2.056	102.700	14.790	0.000
	M	3.191	3.163	2.200	0.410	0.684
Social frequency	U	2.795	2.563	23.400	3.630	0.000
	M	2.781	2.798	−1.700	−0.340	0.736
Social class	U	2.619	2.334	34.100	5.310	0.000
	M	2.579	2.585	−0.700	−0.150	0.882
Social interaction	U	4.319	3.516	45.400	7.360	0.000
	M	4.277	4.304	−1.500	−0.320	0.751
Socioeconomic status	U	2.399	2.225	21.100	3.310	0.001
	M	2.366	2.373	−0.900	−0.190	0.853
Household registration	U	0.391	0.163	52.800	7.640	0.000
	M	0.350	0.383	−7.700	−1.420	0.156

U = unmatched, M = matched. The t-test assesses the equality of covariate means between groups. Smaller absolute *t*-values with *p* > 0.05 after matching indicate satisfactory balance. A displayed *p* = 0.000 represents *p* < 0.001.

Table 6 presents the regression results using the matched sample (*N* = 429). The positive effect of physical exercise on fertility intention remains statistically significant (0.069 or 0.087, *t* = 2.961, *p* < 0.01). This modest attenuation is expected, as PSM removes the overestimation arising from observable differences between exercisers and non-exercisers, and the smaller matched sample yields more conservative estimates. Importantly, the coefficient remains highly significant and the direction is unchanged, confirming that the positive association between physical exercise and fertility intention is not an artifact of selection bias.

**Table 6:** PSM regression results.

Variables	OLS (1)	OLS (2)
	Fertility intention	Fertility intention
Physical exercise	0.083***	0.069***
	(3.280)	(2.961)
Age		0.013***
		(2.739)
Marital status		0.306***
		(3.145)
Educational level		−0.107***
		(−2.992)
Social frequency		0.015
		(0.430)
Social class		0.005
		(0.115)
Social interaction		−0.013
		(−0.637)
Socioeconomic status		0.025
		(0.551)
Household registration		−0.115
		(−1.245)
Constant	1.687***	1.176***
	(37.473)	(4.547)
Observations	429	429
*R*-squared	0.025	0.190
r2_a	0.0223	0.173
*F*	10.76	10.94

*t*-statistics in parentheses; all models are calculated using heteroscedasticity-robust standard errors; ***p < 0.01, **p < 0.05, *p < 0.1.

To address this, we additionally applied kernel matching (bandwidth = 0.06) and radius matching (calipers = 0.01 and 0.05), which retain nearly all observations within the common support region and provide more efficient estimates. As summarized in Table 7, these alternative estimators produced highly consistent and moderately larger effect sizes: the ATT coefficients were 0.142 (kernel), 0.155 (radius, caliper 0.01), and 0.141 (radius, caliper 0.05), all statistically significant at *p* < 0.05. The consistency across these diverse matching specifications, which preserve different sample compositions, substantially strengthens our confidence that the positive association is not an artifact of observable selection bias. Given its efficiency and use of the full common support, we adopt the kernel matching result (ATT = 0.142) as our primary PSM estimate, while transparently noting that 1:1 nearest-neighbor matching yields a more conservative but still significant coefficient (0.069).

**Table 7:** Robustness checks using alternative propensity score matching estimators.

Matching method	Specific settings	ATT coefficient	*t* statistic	Significance
Kernel Matching	Bandwidth = 0.06	0.142	2.19	*p* < 0.05
Radius Matching	Caliper = 0.01	0.155	2.22	*p* < 0.05
Radius Matching	Caliper = 0.05	0.141	2.18	*p* < 0.05
Rosenbaum Bounds	Critical Gamma Value	1.3—1.4(sig^+^ > 0.05)		

ATT = Average Treatment Effect on the Treated. The Rosenbaum bounds critical Γ range (1.3–1.4) indicates the level of unobserved confounding required to overturn the significant nearest-neighbor matching result (at *α* = 0.05). The range arises from slight variations across matching specifications.

To evaluate how sensitive our PSM results might be to unmeasured confounding (i.e., hidden bias), we conducted a Rosenbaum bounds sensitivity analysis for the nearest-neighbor matching specification. As shown in Table 7, the critical Gamma (Γ) values at which the significance level (sig^+^) would exceed 0.05 ranged from 1.3 to 1.4, depending on the model specification. This range implies that an unobserved confounder would need to increase the odds of treatment assignment (i.e., the likelihood of engaging in exercise) by a factor of approximately 1.3 to 1.4 to completely nullify the observed significant effect. Following standard interpretations in observational studies, a Γ value in this range suggests moderate sensitivity to hidden bias. In practical terms, a variable such as unmeasured “health-consciousness”—which likely correlates with both regular exercise and proactive fertility preparation—could plausibly operate at or near this magnitude. Consequently, while our PSM robustness checks rule out confounding by observed covariates, we caution that the inferred association could be partially influenced by unobserved psychosocial factors.

### 3.5 Change the econometric model: Ordered Probit robustness test

To further address concerns that our use of OLS might be inappropriate for an ordered categorical dependent variable, we re-estimated the relationship using an Ordered Probit model. This specification explicitly treats fertility intention (ranging from 0 to 6) as ordered, thereby relaxing the OLS assumption of equal interval spacing between response categories.

As shown in Table 8, the Ordered Probit results are fully consistent with the OLS findings. Physical exercise retains a positive and highly significant effect on fertility intention in both the parsimonious model (coefficient = 0.063, *z* = 2.792, *p* < 0.01) and the full model with controls (coefficient = 0.139, *z* = 5.589, *p* < 0.01). The pseudo *R*^2^ improves from 0.003 to 0.073 with the inclusion of control variables, paralleling the pattern observed in the OLS specification.

**Table 8:** Robustness test results based on Ordered Probit model.

Variables	Ordered Probit (1)	Ordered Probit (2)
	Fertility intention	Fertility intention
Physical exercise	0.063***	0.139***
	(2.792)	(5.589)
Age		0.021***
		(4.495)
Marital status		0.270***
		(2.998)
Educational level		−0.134***
		(−4.261)
Social frequency		0.053
		(1.482)
Social class		0.054
		(1.155)
Social interaction		−0.032
		(−1.449)
Socioeconomic status		0.008
		(0.171)
Household registration		−0.257***
		(−3.315)
Observations	1,230	1,230
Pseudo *R*^2^	0.0030	0.0730

*z*-statistics in parentheses; all models are calculated using heteroscedasticity-robust standard errors; ***p < 0.01, **p < 0.05, *p < 0.1.

In summary, the robustness tests—PSM to address selection bias and Ordered Probit to address functional form concerns—both corroborate the benchmark findings. The positive association between physical exercise and fertility intention is stable across different identification strategies and model specifications, substantially strengthening confidence in the reliability of our core conclusion.

### 3.6 Decomposition of the association: indirect pathways via health and well-being

To investigate the mechanisms through which physical exercise may influence fertility intention, we fitted a parallel mediation model that simultaneously included health status and subjective well-being as mediators. This specification allows us to estimate the unique indirect effect of each mediator while controlling the other, and to test whether the two pathways are complementary rather than redundant.

First, we examined the effects of physical exercise on each mediator (Tables 9, 10). Physical exercise was positively and significantly associated with health status (coefficient = 0.068, *t* = 3.77, *p* < 0.001) and with subjective well-being (coefficient = 0.045, *t* = 3.14, *p* < 0.01). These results confirm that more frequent exercise relates to better self-rated health and higher levels of happiness.

**Table 9:** First-stage regression results: physical exercise predicting health status.

Variables	Overall effects	95% conf. interval (lower_upper)	
Physical exercise	0.068***	0.033	0.104
	(3.77)		
Age	−0.0262***	−0.033	−0.019
	(−7.57)		
Marital status	0.049	−0.082	0.179
	(0.73)		
Education level	−0.0324	−0.078	0.013
	(−1.41)		
Social frequency	0.038	−0.014	0.090
	(1.42)		
Social class	0.125***	0.057	0.194
	(3.60)		
Social interaction	0.030	−0.002	0.061
	(1.85)		
Socioeconomic status	0.027	−0.042	0.096
	(0.76)		
Household registration	0.004	−0.108	0.117
	(0.07)		
Constant	4.180***	3.814	4.546
	(22.40)		

*t*-statistics in parentheses; OLS estimation with heteroskedasticity-robust standard errors; ***p < 0.01, **p < 0.05, *p < 0.1. This table reports the first stage of the mediation analysis (physical exercise → health status). The full mediation results are presented in Table 11.

**Table 10:** First-stage regression results: physical exercise predicting subjective well-being.

Variables	Overall effects	95% conf. interval (lower_upper)	
Physical exercise	0.045***	0.017	0.074
	(3.14)		
Age	−0.006**	−0.011	−0.001
	(−2.17)		
Marital status	0.138***	0.035	0.242
	(2.62)		
Education level	0.014	−0.022	0.050
	(0.76)		
Social frequency	0.030	−0.011	0.071
	(1.42)		
Social class	0.140***	0.086	0.194
	(5.06)		
Social interaction	0.012	−0.013	0.037
	(0.95)		
Socioeconomic status	0.015	−0.040	0.069
	(0.52)		
Household registration	0.017	−0.073	0.106
	(0.36)		
Constant	3.476***	3.186	3.767
	(23.44)		

*t*-statistics in parentheses; OLS estimation with heteroskedasticity-robust standard errors; ***p < 0.01, **p < 0.05, *p < 0.1. This table reports the first stage of the mediation analysis (physical exercise → subjective well-being). The full mediation results are presented in Table 11.

Next, we estimated the full model, in which fertility intention was regressed on physical exercise, health status, subjective well-being, and all covariates (Table 11). In this joint model, both health status (coefficient = 0.066, *t* = 2.81, *p* < 0.01) and subjective well-being (coefficient = 0.087, *t* = 2.94, *p* < 0.01) remained significant independent predictors of fertility intention. Importantly, each mediator retained its significance after controlling for the other, indicating that they contribute incrementally to fertility intention.

**Table 11:** Bootstrap mediation effect test results (*N* = 1,230, Resamplings = 1,000).

Point estimate	Overall effects	95%CI Lower	95%CI Upper	% of total effect
FI				
Health status	0.066(2.81) ***	0.020	0.113	
Subjective well-being	0.087(2.94) ***	0.029	0.146	
Total indirect effect (a₁b₁ + a₂b₂)	0.0085(3.31) ***	0.0035	0.0135	9.7
Indirect effect via health (a₁b₁)	0.0045(2.25) **	0.0006	0.0085	4.6
Indirect effect via well-being (a₂b₂)	0.0040(2.15) **	0.0003	0.0076	5.2
Direct effect (c′)	0.0787(5.30) ***	0.0496	0.1078	90.3
Total effect (c)	0.0872(5.88) ***	0.0581	0.1163	100
cov (e. Health, e. Happy)	0.1138(6.95) ***	0.0817	0.1459	

Bootstrap percentile CIs. **p < 0.05, ***p < 0.01; *z*-statistics in parentheses; all models estimated using heteroskedasticity-robust standard errors. c = total effect; c′ = direct effect; a × b = indirect effect.

The total effect of physical exercise on fertility intention was *c*′ = 0.0872 (*p* < 0.001). When the two mediators were included, the direct effect dropped to *c*′ = 0.0787 (*p* < 0.001), suggesting partial mediation. The specific indirect effects were computed as the products of the respective paths: Via health status: *a*₁ × *b*₁ = 0.068 × 0.066 = 0.0045; Via subjective well-being *a*₂ × *b*₂ = 0.045 × 0.087 = 0.0039; Total indirect effect = 0.0085.

Notably, the residual covariance between health status and subjective well-being was statistically significant (cov = 0.114, *p* < 0.001), indicating that these two mediators share common unobserved determinants. This finding further justifies the parallel mediation specification and supports the interpretation that the two pathways are complementary—each capturing a distinct set of processes (physiological health vs. psychological well-being) through which exercise may enhance fertility intentions.

Taken together, these stepwise results provide initial support for Hypotheses H2 and H3, demonstrating that both health status and subjective well-being partially mediate the positive association between physical exercise and fertility intention.

### 3.7 Bootstrap assessment of indirect pathways

To overcome the limitations of the normal-theory approach and to obtain robust standard errors for the indirect effects, we employed bootstrap resampling with 1,000 replications. The parallel mediation model showed to the data (RMSEA = 0.000, CFI = 1.000, SRMR = 0.000), indicating that the saturated model perfectly reproduced the observed covariance structure.

As displayed in Table 11, the bootstrap 95% confidence intervals for the indirect effects excluded zero: Indirect effect via health status: 0.0045 (95% CI [0.0006, 0.0085]); Indirect effect via subjective well-being: 0.0040 (95% CI [0.0003, 0.0076]); Total indirect effect: 0.0085 (95% CI [0.0035, 0.0135]).

The direct effect (*c*′ = 0.0787) remained significant (95% CI [0.0496, 0.1078]), confirming that the mediation is partial rather than full. The significant residual covariance between the two mediators (cov = 0.114, *p* < 0.001) reinforces that they are correlated yet not redundant—each represents a distinct pathway through which physical exercise may promote fertility intention.

To quantify the explanatory power of the mediators, we calculated the proportion of the total effect accounted for by each indirect pathway. The total indirect effect (0.0085) represents 9.7% of the total effect (0.0872). Specifically, health status accounts for 5.2% (0.0045/0.0872) and subjective well-being accounts for 4.6% (0.0040/0.0872). The direct effect (0.0787) accounts for the remaining 90.3%. Although the bootstrap 95% confidence intervals for both indirect effects exclude zero, the lower bound for the health pathway (0.0006) is close to zero, indicating that the indirect effect is small and estimated with limited precision.

These bootstrap results corroborate the stepwise mediation findings and provide robust statistical evidence that both health status and subjective well-being serve as complementary partial mediators. Thus, Hypotheses H2 and H3 are strongly supported.

### 3.8 Heterogeneity analysis

To examine whether the promotional effect of physical exercise on fertility intention varies across demographic subgroups, we conducted heterogeneity analyses by age group and urban–rural residence. Rather than simply comparing coefficients across separate subsamples—a practice that can be statistically misleading—we estimated pooled regressions with interaction terms, which directly test whether the coefficient differences are statistically significant. Table 12 presents the full regression results. To ensure consistency across specifications, the same control variable system was used as in the baseline regression. For the urban–rural grouped regression, the coefficient of household registration is not reported due to perfect multicollinearity with the grouping variable, but the variable was controlled for in the age-grouped regressions.

**Table 12:** Heterogeneity analysis: subgroup regressions and formal interaction tests.

Variables	Age		Urban and rural areas	
	(≤30 years old)	(>30 years old)	(Rural)	(Urban)
	Fertility intention	Fertility intention	Fertility intention	Fertility intention
Physical exercise	0.106***	0.084***	0.077***	0.114***
	(2.942)	(5.182)	(4.680)	(3.568)
Age	0.029**	0.011***	0.012***	0.013**
	(2.031)	(2.860)	(3.524)	(2.507)
Marital status	0.214*	0.150**	0.155**	0.207**
	(1.794)	(2.038)	(2.310)	(2.097)
Education level	−0.035	−0.098***	−0.076***	−0.087**
	(−0.809)	(−4.578)	(−3.316)	(−2.464)
Social frequency	0.081*	0.006	0.035	0.001
	(1.722)	(0.258)	(1.414)	(0.026)
Social class	0.041	0.028	0.015	0.066
	(0.649)	(0.890)	(0.482)	(1.109)
Social interaction	−0.042	−0.012	−0.024*	−0.010
	(−1.333)	(−0.870)	(−1.666)	(−0.338)
Socioeconomic status	0.030	−0.009	0.018	−0.033
	(0.502)	(−0.286)	(0.543)	(−0.561)
Household registration	−0.277***	−0.086		
	(−3.087)	(−1.525)		
Constant	0.485	1.377***	1.267***	1.002***
	(1.127)	(6.407)	(6.832)	(3.229)
Observations	349	881	822	408
*R*-squared	0.121	0.092	0.125	0.127
Adjusted R-squared	0.0978	0.0825	0.116	0.110
*F*	5.190	9.790	14.52	7.286

Interaction Term	Coefficient	*t*	*F*	*p*-value
PE × Age >30	−0.025	−0.73	0.53	0.465
PE × Urban	0.036	1.13	1.27	0.260

*t*-statistics in parentheses; robust standard errors; *p < 0.1, **p < 0.05, ***p < 0.01. Interaction terms were estimated in separate pooled OLS regressions including all covariates shown above. Age ≤30 (vs. >30) and Rural (vs. Urban) were used as reference groups, respectively. *F*-tests for the addition of interaction terms: *F*(1, 1,218) = 0.53, *p* = 0.465 (age); *F*(1, 1,218) = 1.27, *p* = 0.260 (residence).

The effect of physical exercise on fertility intention is more pronounced among women aged ≤30 years, with a regression coefficient of 0.106 (*t* = 2.942, *p* < 0.01) compared to 0.084 (*t* = 5.182, *p* < 0.01) for women aged>30 years. The effect is also more pronounced among urban women, with a coefficient of 0.114 (*t* = 3.568, *p* < 0.01), compared to 0.077 (*t* = 4.680, *p* < 0.01) for rural women.

As presented in Table 12, the interaction term for age (Physical Exercise × Age<30) yielded a coefficient of −0.025 (*t* = −0.73, *p* = 0.465), indicating no statistically significant difference between the two age groups. The *F*-test for the age-interaction term was 0.53 (*p* = 0.465). Similarly, the interaction term for residence (Physical Exercise × Urban) was 0.036 (*t* = 1.13, *p* = 0.260), with an F-test of 1.27 (*p* = 0.260), also failing to reach conventional significance levels.

Descriptively, the coefficients are larger for younger and urban women, but the interaction terms are not statistically significant (*p* > 0.05), indicating that the sample does not provide sufficient evidence for differential effects across these subgroups. From a practical perspective, targeted fitness interventions for young urban women may still deliver relatively larger fertility promotion gains; yet we cannot rigorously conclude that the causal impact of physical exercise systematically differs across age or urban–rural groups based on the current sample evidence.

## 4 Discussion

This study empirically examined the influence of physical exercise on fertility intentions among Chinese women of childbearing age, investigating the mediating roles of health status and subjective well-being, as well as heterogeneity by age and urban–rural residence. Although prior research has examined associations between healthy lifestyles and fertility, few studies have systematically elucidated the specific pathways through which physical exercise affects fertility intention. Our findings contribute to the literature by providing empirical evidence from a large, nationally representative sample and by clarifying the dual mediation mechanisms.

Before interpreting the mediating pathways, we must acknowledge a measurement constraint. Physical exercise was assessed by a single frequency item, without distinguishing intensity, duration, or type; health status and subjective well-being were each measured by a single five-point self-report. These global proxies are suitable for detecting broad correlational patterns, but they cannot empirically validate specific physiological or neuroendocrine processes (e.g., hormone kinetics, cortisol, endorphins). Consequently, any reference to such mechanisms in the following discussion should be understood as speculative, hypothesis-generating interpretation drawn from external literature, not as direct explanations of our estimated coefficients.

### 4.1 The positive association between physical exercise and fertility intention

Our benchmark regression results consistently reveal a significant positive association between physical exercise and fertility intention among women of childbearing age (coefficient = 0.087, *p* < 0.01), a finding corroborated by PSM and Ordered Probit robustness checks. This positive association aligns with Leibenstein’s (10, 29) theoretical perspective that non-economic factors—particularly those enhancing individual well-being—play a substantial role in fertility decision-making, and extends the second demographic transition framework by identifying physical exercise as a concrete behavioral pathway through which shifting value orientations toward self-fulfillment may nonetheless coexist with reproductive intentions. From a policy perspective, our findings are hypothesis-generating rather than prescriptive. The observed positive association between physical exercise and fertility intention does not, by itself, warrant direct policy intervention aimed at increasing exercise to raise fertility rates—particularly given the possibility of reverse causation (i.e., women with stronger childbearing desires may proactively engage in preconception health behaviors, including exercise). Instead, the results suggest two more modest policy-relevant implications: First, existing public health infrastructure that promotes physical activity (e.g., community fitness programs, accessible sports facilities) may serve dual purposes—improving general population health while potentially creating conditions conducive to reproductive health. This is consistent with ongoing health promotion efforts and does not require new policy instruments. Second, fertility support programs could consider including physical health and well-being components as part of comprehensive preconception care, rather than treating exercise as a standalone fertility-promoting intervention. Such programs should be evaluated through pilot studies with longitudinal designs before scaling up. Crucially, we caution against interpreting our estimates as implying that increasing exercise frequency in the population would mechanically raise aggregate fertility rates. The modest effect sizes (coefficient ≈0.07–0.09 on a 0–6 scale) and the partial mediation pathways suggest that any population-level impact would likely be small and context-dependent. From a physiological standpoint, regular exercise has been speculated to regulate endocrine function, improve cardiorespiratory fitness, and alleviate chronic stress, thereby potentially establishing a healthy physiological foundation conducive to conception and pregnancy (15). From a social support perspective, exercise facilitates the formation of extensive social networks through group activities, reducing the isolation often associated with parenting and transforming social capital into shared resources that lower perceived parenting costs. Culturally, women who actively engage in exercise are often perceived as embodying health and vitality, which may enhance their social standing and self-confidence, indirectly strengthening fertility willingness. As physical exercise becomes emblematic of a fertility-friendly lifestyle, its modeling effect may gradually reshape cultural perceptions of childbearing among women of reproductive age. However, the relationship between exercise and fertility is not universally positive across contexts. Zhao et al.’s (35) meta-analysis of European subgroups found no significant association between moderate-intensity exercise and fertility, while high-intensity exercise was significantly negatively correlated, suggesting that the influence of physical exercise on fertility may be moderated by welfare system characteristics, cultural norms, and baseline physical activity levels. This contextual variability underscores the importance of situating our findings within China’s specific institutional and cultural landscape, where public fitness infrastructure is rapidly expanding and fertility policies are actively evolving.

### 4.2 Potential pathways consistent with the data: health and well-being as statistical mediators

Our parallel mediation analysis, which simultaneously estimates both pathways, reveals that health status and subjective well-being serve as independent partial mediators of the exercise-fertility relationship, each retaining significance after controlling for the other, with indirect effects of 0.0045 (95% CI: 0.0006–0.0085) and 0.0040 (95% CI: 0.0003–0.0076). The total indirect effect via both pathways was 0.0085.

The health pathway operates through physiological improvements. Our results indicate that exercise significantly improves self-rated health (coefficient = 0.068, *p* < 0.01), which in turn positively predicts fertility intention (coefficient = 0.066, *p* < 0.01) in the parallel mediation model that also accounts for subjective well-being. This is consistent with the view that regular exercise optimizes cardiopulmonary function, regulates endocrine balance, and alleviates chronic pain—all of which provide a physiological basis for women to bear the physical costs of pregnancy and childbirth, thereby reducing health-related anxieties about fertility. When women perceive their bodies as capable and resilient, their confidence in conceiving and carrying a pregnancy to term increases, translating into stronger fertility intentions. Comparing the two pathways, the indirect effect via health status (0.0045) is marginally larger than that via subjective well-being (0.0040). However, the more critical finding is that both mediators retain independent significance when entered simultaneously, and their residual covariance is statistically significant (cov = 0.114, *p* < 0.001). This covariance pattern suggests that although the two mechanisms are correlated—likely sharing common antecedents such as lifestyle coherence or personality traits—they are not redundant. Rather, they capture complementary dimensions: one operating through physiological readiness and the other through positive psychological appraisal. This complementarity reinforces the theoretical view that physical exercise enhances fertility intention through a holistic mind–body pathway. This aligns speculatively with the neurobiochemical understanding that exercise-induced endorphin release alleviates stress hormones, including cortisol, thereby reducing anxiety and depression (36). The sense of accomplishment, mastery, and belonging derived from regular physical activity may foster a generalized sense of self-efficacy that extends to the parenting domain—an accumulation of psychological capital that transforms perceived parenting responsibilities from an overwhelming burden into a manageable life project. When women of childbearing age experience positive emotions such as vitality and social connectedness through exercise, their subjective well-being increases, effectively lowering the threshold at which fertility intentions are activated, even under identical objective conditions (37). This physiological-psychological feedback loop—wherein exercise is related to health, which stimulates positive emotions, which in turn sustain healthy behaviors—represents a crucial bridge from physical practice to reproductive consciousness.

It is important to note that the two mediators together accounted for less than 10% of the total association. This suggests that much of the exercise-ideal fertility association operates through other, unmeasured pathways, such as partner dynamics, social support, or broader lifestyle factors. Therefore, while our findings are consistent with health and well-being playing a small, complementary role, the primary mechanisms remain to be explored in future research. Zhang et al. (38), drawing on a sample of 2,796 women from NHANES 2013–2020, found a non-linear relationship between exercise and infertility, while Wise et al. (39) confirmed heterogeneous effects by activity intensity, suggesting dose-effect boundaries and activity-specific conditions that our ordinal frequency measure could not capture. Additionally, factors such as age, socioeconomic status, and family support environment may modulate both the conversion efficiency of exercise into health capital and its subsequent effects on well-being and fertility intentions. Thus, while the dual mediation framework is theoretically and empirically defensible, the underlying mechanisms are likely more nuanced than our global measures can fully reveal.

### 4.3 Heterogeneity: age and urban–rural differences

Although our formal interaction tests did not yield statistically significant subgroup differences (*p* = 0.465 for age; *p* = 0.260 for residence), we observe descriptive patterns indicating higher values among younger women and urban women. These observations may be worth exploring in future hypothesis-generating research. With this caveat, we tentatively offer the following speculative interpretations, which await empirical validation through longitudinal or experimental designs.

#### 4.3.1 Age-based heterogeneity

Physical exercise has positive effect on fertility intention among both women aged ≤ 30 and women > 30, with a stronger promoting effect observed in the group aged ≤30, a pattern consistent with life-course dynamics. Younger women are typically in the early stages of marriage and within their optimal reproductive window, which might speculatively make them more physiologically responsive to exercise-induced improvements in endocrine function, including estrogen regulation and ovarian function optimization (13), the perceptible improvements in physical conditioning at this life stage can markedly boost confidence in conception and foster positive expectations toward reproductive behavior. Moreover, younger women generally face lighter parenting burdens and can more readily integrate exercise into their daily routines, enhancing marital satisfaction and subjective well-being through shared physical activities. For women over 30, particularly those who already have one or more children, parenting responsibilities increasingly compete for time and energy; exercise habits, if maintained, tend to shift toward health maintenance rather than fertility preparation, yielding diminishing marginal effects on fertility intention.

#### 4.3.2 Residence-based heterogeneity

Physical exercise exhibits a statistically significant positive effect on fertility intention among both rural and urban samples. Nevertheless, the magnitude of the coefficient is larger for the urban group, indicating that the facilitating effect tends to be stronger among urban women. A finding that likely reflects differential access to sports infrastructure and social resources. Speculatively, urban women are more likely to engage in group-based sports activities, through which they can establish social support networks, exchange fertility-related information, and receive peer encouragement—all of which may mitigate fertility anxiety and indirectly boost fertility confidence. In contrast, rural women often engage in more individualized physical activities, and the relative scarcity of sports facilities and organized programs reduces the conversion efficiency of exercise into fertility-relevant psychological and social capital. It is worth noting, however, that heterogeneity extends beyond age and residence. Li et al. (40) found that higher education significantly reduced fertility intentions among women but not men, a pattern attributed to the time-squeeze effect of career development. Similarly, from an economic standpoint, low-income women face a double constraint—limited access to sports facilities and tighter time budgets—which may weaken the positive effect of exercise on fertility decisions. These observations suggest that the promotional effect of physical exercise on fertility intention is multi-dimensional and moderated by a constellation of demographic and socioeconomic factors, reinforcing the need for targeted, subgroup-sensitive policy interventions.

However, because the interaction terms were not statistically significant, these interpretations should be viewed strictly as exploratory. Future studies with larger samples and more precise measures of exercise type, intensity, and duration are needed to determine whether age- or residence-specific effects truly exist.

### 4.4 Limitations of the study

First, our measure of fertility intention captures ideal rather than planned childbearing, which may diverge from actual behavior. Future research should adopt more behaviorally proximate indicators, such as expected fertility within a specific timeframe. Second, the data from this study cannot clarify the chronological sequence of the variables. The reverse causality is a highly persuasive alternative explanation: Women with a stronger desire to have children tend to actively engage in pre-pregnancy health management behaviors, including exercise. Third, our mediators—self-rated health and subjective well-being—are global proxies that cannot pinpoint specific physiological or psychological pathways. Future work incorporating objective biomarkers (e.g., hormonal profiles) and domain-specific psychological measures (e.g., fertility-related anxiety) would strengthen mechanistic understanding. Fourth, our heterogeneity analysis was limited to age and residence. Other potential moderators—education, income, parity—warrant systematic examination in future studies. Fifth, although we employed multiple PSM estimators to reduce observable selection bias, the Rosenbaum sensitivity analysis (critical Γ ranging from 1.3 to 1.4) indicates that our findings are moderately sensitive to unmeasured confounding. Unobserved characteristics such as personality traits, health consciousness, or partner support could potentially attenuate the estimated association if they strongly influence both exercise participation and fertility intentions. We therefore interpret the PSM results as providing suggestive evidence of association rather than definitive causal identification. Future longitudinal or instrumental-variable designs are needed to more rigorously address endogeneity.

## 5 Conclusion

This study provides preliminary, associational evidence that physical exercise correlates with higher fertility intentions. This association operates through two distinct and complementary mediation pathways—health status and subjective well-being—as confirmed by a parallel mediation model. The relationship shows descriptively larger coefficients among younger and urban women, but these subgroup differences are not statistically confirmed in our data. These findings suggest that physical exercise merits further investigation as a potential low-cost, non-economic adjunct, but policy implementation requires evidence from interventional or quasi-experimental studies.

## Data Availability

The original contributions presented in the study are included in the article/supplementary material, further inquiries can be directed to the corresponding author.
